# aeGEPUCI: a database of gene expression in the dengue vector mosquito, *Aedes aegypti*

**DOI:** 10.1186/1756-0500-3-248

**Published:** 2010-10-04

**Authors:** Sumudu N Dissanayake, Jose MC Ribeiro, Mei-Hui Wang, William A Dunn, Guiyun Yan, Anthony A James, Osvaldo Marinotti

**Affiliations:** 1 Department of Molecular Biology and Biochemistry, University of California, Irvine, CA 92697, USA; 2 Laboratory of Malaria and Vector Research, National Institutes of Health (NIH/NIAID), Rockville, MD 20852, USA; 3 Program in Public Health, University of California, Irvine, CA 92697, USA; 4 Department of Microbiology and Molecular Genetics, University of California, Irvine, CA 92697, USA

## Abstract

**Background:**

*Aedes aegypti *is the principal vector of dengue and yellow fever viruses. The availability of the sequenced and annotated genome enables genome-wide analyses of gene expression in this mosquito. The large amount of data resulting from these analyses requires efficient cataloguing before it becomes useful as the basis for new insights into gene expression patterns and studies of the underlying molecular mechanisms for generating these patterns.

**Findings:**

We provide a publicly-accessible database and data-mining tool, aeGEPUCI, that integrates 1) microarray analyses of sex- and stage-specific gene expression in *Ae. aegypti*, 2) functional gene annotation, 3) genomic sequence data, and 4) computational sequence analysis tools. The database can be used to identify genes expressed in particular stages and patterns of interest, and to analyze putative *cis*-regulatory elements (CREs) that may play a role in coordinating these patterns. The database is accessible from the address http://www.aegep.bio.uci.edu.

**Conclusions:**

The combination of gene expression, function and sequence data coupled with integrated sequence analysis tools allows for identification of expression patterns and streamlines the development of CRE predictions and experiments to assess how patterns of expression are coordinated at the molecular level.

## Findings

The completed sequence of the *Ae. aegypti *genome [1] has enhanced the development of novel methods of manipulating vector populations to effect control of disease transmission [2]. In order to further the prospects of such endeavours, we generated and organized data using gene expression microarrays to quantify genome-wide transcription in adult males and females in different developmental stages. Adult male and female mosquitoes feed on sugar obtained mostly from nectar of flowers and honeydew to meet the energy demands of basal metabolism and flight. In addition, female mosquitoes also feed on blood for egg development. Since this behaviour is associated with reproduction and disease transmission, our study explored changes in gene expression following a blood meal. Arrangement of these data into a searchable format has streamlined the elucidation of those genes that are expressed in a stage- and sex-specific/enhanced manner. In addition, by integrating DNA sequence comparison tools with a pattern-finding interface, analyses of putative *cis*-regulatory elements (CREs) can be performed on sets of genes that share similar patterns of expression. Building upon our foundation of the *Anopheles gambiae *Gene Expression Profile at UC Irvine, http://www.angaged.bio.uci.edu, [3-5], a study of an African vector of human malaria, we provide here a public database and web-based data-mining tool that combine staged expression microarray data, functional annotation, genomic sequence data, and integrated DNA sequence comparison algorithms to gain insight into gene expression and regulation in *Ae. aegypti*.

### Data collection

**Figure 1:**
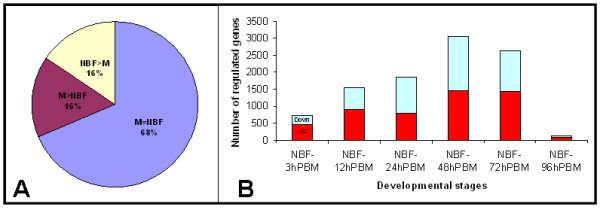
**Sex-biased and bloodmeal-induced gene expression in *Aedes aegypti***. **A- **Representation of sex-biased gene expression in adult *Ae. aegypti*. A total of 5,081 (32%) transcripts are accumulated at different levels (p-values < 0.001) between males (M) and non blood-fed females (NBF) (M> NBF, 2557 transcripts and NBF > M, 2524 transcripts), while 11,141 (M = NBF, 68%) did not show significant difference in accumulation between the two sexes. **B- **Numbers of *Ae. aegypti *transcripts up- or down-regulated significantly (p-values < 0.001) in females at 3, 12, 24, 48, 72, and 96 hours post bloodmeal (PBM). A total of 4,773 (30%) transcripts were found to vary in accumulation in females following a blood meal. The numbers of varying transcripts were calculated by comparison of each sample with four-day old non blood-fed females and depicted separately as up-regulated (red) and down-regulated (blue). A Bayesian t-test was performed to calculate p-values using the statistical analysis package Cyber-T at http://cybert.microarray.ics.uci.edu/.

Stage-specific transcriptional signal values were imported from genome-wide microarray analyses of four-day old adult males, four-day old nonblood-fed adult females, and blood-fed adult females at 3, 12, 24, 48, 72, and 96 hours after a bloodmeal. RNA was extracted from whole mosquitoes and hybridized according to standard protocols to custom-designed microarrays that survey 16,222 *Ae. aegypti *transcripts (Platform GPL10542). Expression signals were normalized for background within chips with the Agilent spatial correction algorithm (gprocessed signals) and analyzed with JMP Genomics software http://www.jmp.com/software/genomics/ and Cyber-T http://cybert.microarray.ics.uci.edu/. Microarray data and detailed experimental protocols have been submitted to the Gene Expression Omnibus under the accession series: GSE22339. A total of 5,081 (32%) transcripts were identified as having sex-specific or preferential expression (p-values < 0.001). Of those genes exhibiting sex-differential expression, 2,557 accumulated transcripts at higher levels in males, while the remaining 2,524 were found preferentially or specifically in females (Figure 1A). Extensive variation in gene expression was observed in blood-fed females. A total of 4,773 transcripts were found to vary (p-values < 0.001) in accumulation during oogenesis in at least one of the analysed experimental time points when compared with nonblood-fed females (Figure 1B). Several transcripts, such as those corresponding to members of the D7-related group (AAEL006424-RA, AAEL007394-RB) and an apyrase (AAEL006347-RA), are expressed preferentially in adult female salivary glands and assist blood ingestion [6]. Accordingly, these were detected as female-enhanced in our dataset. Similarly, transcripts characterized previously as accumulating in female mosquitoes following a blood meal, such as those involved with digestion (Late Trypsin, AAEL013284-RA) [7] or reproduction (Vitellogenin, AAEL006126-RA) [8], also were determined in our dataset to be upregulated after blood ingestion, supporting the legitimacy of the dataset presented here.

Functional gene annotation was imported from the AegyXcel database http://exon.niaid.nih.gov/transcriptome.html#aegyxcel to populate aeGEPUCI with keywords and annotation from the ENSEMBL, NCBI non-redundant, GO, PFAM, and SMART databases. Putative promoter sequences were selected as regions 2.0 kilobases (kb) in length immediately adjacent to the 5'-ends of the annotated 5' untranslated regions (UTRs) where available, or 5'-ends of coding sequences using genomic data from Vectorbase.org (Assembly: AaegL1, Oct 2005; Genebuild: VectorBase, Aug 2006; Database version: 55.1d).

### Implementation

**Figure 2:**
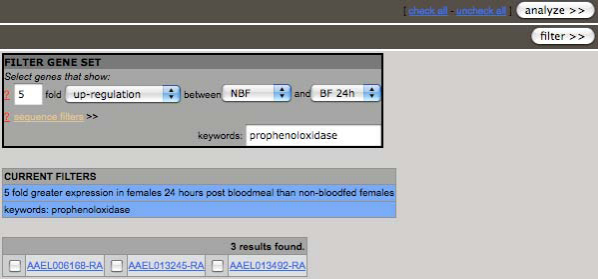
**Data-mining interface**. The "Filter gene set" data-mining interface allows users to select a gene set that meets specific expression, keyword, and sequence criteria. Input fields include a) differential expression quantified from stage-specific expression microarray analyses, b) keywords included in functional annotation gathered by AegyXcel http://exon.niaid.nih.gov/transcriptome.html#aegyxcel from the ENSEMBL, NCBI non-redundant, PFAM, GO, and SMART databases, and c) DNA sequences contained within promoter, 5' untranslated region, 3' untranslated region, or cDNA sequences from the *Ae. aegypti *genome. Each filter is imposed on the current gene set being examined, beginning with the entire *Ae. aegypti *genome, thus selecting and reducing the gene set in a stepwise fashion as genes matching previous filter criteria are eliminated by subsequent filters. The parameters and gene set shown here are those corresponding to the prophenoloxidase case study described in the text.

The data have been stored as a MySQL relational database that is accessible directly through an Apache web server http://www.apache.org. A web-based data-mining interface is used to manage queries to identify genes that meet specific expression, keyword, and sequence criteria (Figure 2). Sequence comparison and motif-finding tools, including MEME [9] and AlignACE [10,11], are accessible directly through the data-mining interface for analysis of CREs corresponding to a selected gene set.

### Data retrieval

**Figure 3:**
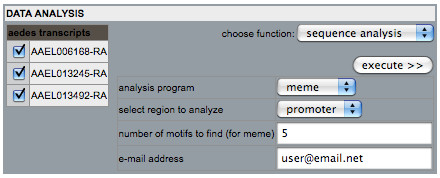
**Data analysis menu**. The three transcripts comprising the prophenoloxidase case study gene set are shown in the data analysis menu. The functional drop-down menu allows users to select their desired analysis function: display expression profiles in batch, export genomic sequences in FASTA format, or perform sequence analysis. To perform sequence analysis, an analysis program (MEME or AlignACE) and desired region to analyze (promoter, 5' UTR, 3' UTR, or cDNA) are chosen from the drop-down menus; MEME and promoters, respectively, in this case. Analysis programs are run using default parameters. If MEME is chosen as the desired analysis program, the number of motifs to search for can be specified, with five chosen in this example. AlignACE does not require specification of the number of motifs for which to search. An e-mail address may be specified for automatic notification once analysis is complete and results are ready for viewing.

The data-mining interface is accessible from the main page of the site and allows users to focus on specific genes that satisfy desired criteria based on: 1) stage- and sex-specific expression, 2) annotated keywords, or 3) DNA sequences in promoter, 3' UTR, 5' UTR, or cDNA (Figure 2). The data-mining tool allows users to arrive at an increasingly specific gene set by imposing in a stepwise fashion each new filter criterion upon all preceding criteria. After selecting a set of genes of interest, users can access the analysis menu to perform CRE analyses, view expression profiles in batch, or export associated DNA sequences (Figure 3). Detailed annotation and expression data for each gene also can be viewed by selecting the gene-identifier link to open the description of a gene entry.

### Description of a gene entry

**Figure 4:**
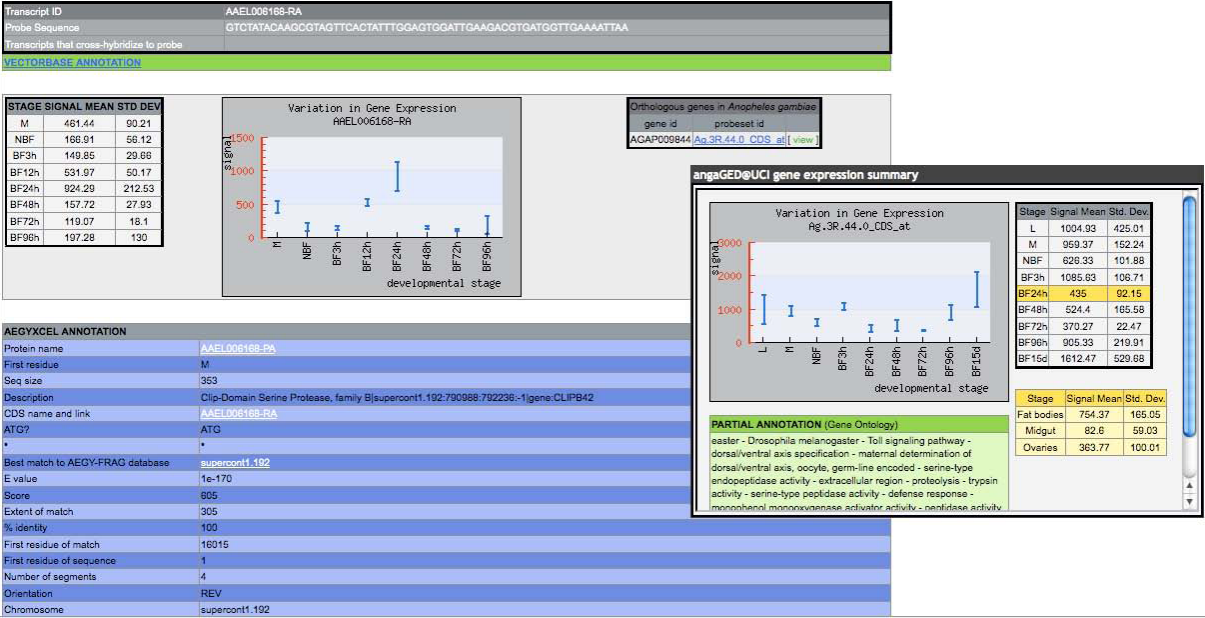
**Gene entry for one transcript**. Abbreviated gene description for one transcript, AAEL006168-RA. Each entry displays the developmental expression profile built for the transcript from stage-specific microarray analyses, the probe sequence and any other transcripts that cross-hybridize to this probe, alongside a link to Vectorbase http://www.vectorbase.org and functional annotation gathered by AegyXcel http://www.anobase.org/genes/AegyXcel.html from the ENSEMBL, NCBI non-redundant, PFAM, GO, and SMART databases. If present, orthologous genes are displayed from the *Anopheles gambiae *Gene Expression Profile at UC Irvine [3] as an inset on the right, with *An. gambiae *probeset identifier Ag.3R.44.0_CDS_at shown here as an example.

Each gene in the *Ae. aegypti *genome has a corresponding data page that may be accessed by selecting the gene-identifier link during data retrieval. Gene entry pages display microarray expression values and functional annotation as gathered by AegyXcel from ENSEMBL, NCBI non-redundant, GO, PFAM, and SMART databases (Figure 4). A link to Vectorbase http://www.vectorbase.org on each entry page provides access to additional, centralized gene data. Furthermore, any corresponding links to orthologous genes in the *Anopheles gambiae *Gene Expression Profile at UC Irvine [3] are provided as a bridge between these two mosquito species.

### Analyzing putative CREs associated with of a gene set of interest

After identifying gene sets that show similar patterns of expression, users can use the menu to directly execute analyses to identify corresponding putative CREs in the set (Figure 3). The interface allows searching for conserved motifs in regulatory domains within UTRs and protein-encoding sequences. Programs currently available for sequence analysis include MEME and AlignACE, which are run using default parameters. MEME allows users to specify the number of motifs for which to search. AlignACE does not require this parameter. Users are allowed to enter their e-mail addresses to receive an e-mail notification when their analysis job has completed and is available for viewing.

### Visualization of transcription profiles

**Figure 5:**
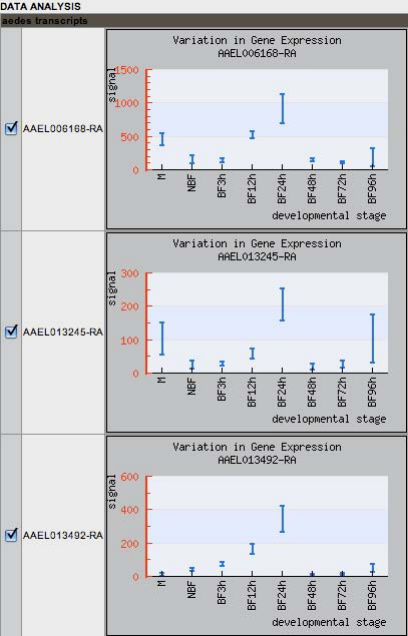
**Developmental expression profiles**. Gene expression profiles measuring transcriptional signal values from stage-specific microarray analyses of the three prophenoloxidase case-study transcripts. The stages shown are male (M), nonblood-fed adult female (NBF), and blood-fed adult female 3, 12, 24, 48, 72, and 96 hours after bloodmeal (BF3h-BF96h).

The analysis menu also allows users to view the transcription profiles of all genes in a gene set in batch. Resulting graphs print gene-expression profiles for all genes in the set according to developmental stage: sugar-fed four-day old adult males, four-day old nonblood-fed adult females, and blood-fed adult females at 3, 12, 24, 48, 72, and 96 hours after a bloodmeal (Figure 5).

### Import gene set

The import gene set link can be used to load a set of gene or transcript identifiers into the data-mining interface for viewing and analysis in batch. This feature allows the application of sequence analysis tools to gene sets built in contexts and with concepts different from the ones utilized in this database. Similarly, gene sets also can be exported from the data-mining interface by using the analysis menu, easing the transfer of the selected genes or sequences for analysis by additional methods.

### Submit a microarray study

The aeGEPUCI database has the capacity to house, integrate, and display additional microarray studies that examine gene expression in *Ae. aegypti*. The Submit Study link provides a form for uploading microarray data and specifications for review and possible integration into the database.

## Utility and Discussion

The aeGEPUCI database identifies genes co-expressed in similar patterns and incorporates keyword searching and sequence analysis into one unified data-mining tool. A case study best illustrates the utility of this integration. In this example, we identify genes linked to the complex regulation of phenoloxidase, an enzyme involved in the melanization of invading parasites and micro-organisms as part of invertebrate innate immunity [12]. Specifically, we search for pro-phenoloxidase genes that are highly expressed 24 hours after bloodfeeding. Two filters are used to complete this inquiry (Figure 2). The first filter selects genes that contain the keyword "prophenoloxidase" in their functional annotation. Seventy-five of the 16,221 transcripts in the *Ae. aegypti *genome contain this keyword. Second, a stage-specific filter identifies 3 of these 75 transcripts that show 5-fold up-regulated expression 24 hours after bloodfeeding (BF24h) as compared to nonblood-fed mosquitoes (NBF).

**Figure 6:**
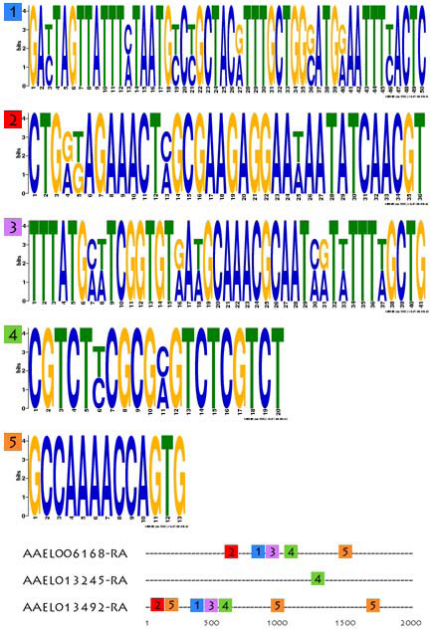
**Sequence analysis and putative CRE discovery**. Conserved sequences corresponding to putative CREs found by MEME and generated through the data analysis menu for the three prophenoloxidase case-study transcripts. Five conserved motifs are displayed as sequence LOGOs, with relative positions mapped in 2.0 kilobases long putative promoter regions shown below (not to scale, length in base-pairs).

The analysis menu can be used to search the 5'-flanking putative promoter regions of genes in this gene set for conserved DNA sequence motifs. Analysis of the promoter regions of the three prophenoloxidase-related genes using MEME shows the occurrence of multiple conserved DNA sequence motifs organized in similar order in putative promoter regions (Figure 6).

In addition to its current microarray data, aeGEPUCI has been built with the goal of expanding its scope to house, integrate, and display information from additional gene expression studies of *Ae. aegypti*. This flexibility assures that aeGEPUCI is capable of growing alongside the increasing quantity of data being produced from other studies. By working closely with Vectorbase http://www.vectorbase.org and other laboratories in this way, it is hoped that aeGEPUCI will act as a catalyst in accelerating the study and understanding of gene expression and regulation in this important vector of disease.

## Availability

The *Aedes aegypti *Gene Expression Profile at UCI is publicly accessible from the URL: http://www.aegep.bio.uci.edu. Questions and comments are welcomed through the site.

## Competing interests

The authors declare that they have no competing interests.

## Authors' contributions

SND designed and implemented the website, database, and wrote the manuscript. OM designed, conducted and analyzed the microarray experiments and wrote the manuscript. MHW analyzed and normalized the microarray data for entry into the database. WAD analyzed data and edited the manuscript. JMCR constructed the AegyXcel database and edited the manuscript. AAJ and GY assisted in the editing of the manuscript. All authors read and approved the final manuscript.

## References

[B1] Nene V Wortman JR Lawson D Haas B Kodira C Tu ZJ Loftus B Xi Z Megy K Grabherr M Ren Q Zdobnov EM Lobo NF Campbell KS Brown SE Bonaldo MF Zhu J Sinkins SP Hogenkamp DG Amedeo P Arensburger P Atkinson PW Bidwell S Biedler J Birney E Bruggner RV Costas J Coy MR Crabtree J Crawford M Debruyn B Decaprio D Eiglmeier K Eisenstadt E El-Dorry H Gelbart WM Gomes SL Hammond M Hannick LI Hogan JR Holmes MH Jaffe D Johnston JS Kennedy RC Koo H Kravitz S Kriventseva EV Kulp D Labutti K Lee E Li S Lovin DD Mao C Mauceli E Menck CF Miller JR Montgomery P Mori A Nascimento AL Naveira HF Nusbaum C O'leary S Orvis J Pertea M Quesneville H Reidenbach KR Rogers YH Roth CW Schneider JR Schatz M Shumway M Stanke M Stinson EO Tubio JM Vanzee JP Verjovski-Almeida S Werner D White O Wyder S Zeng Q Zhao Q Zhao Y Hill CA Raikhel AS Soares MB Knudson DL Lee NH Galagan J Salzberg SL Paulsen IT Dimopoulos G Collins FH Birren B Fraser-Liggett CM Severson DW Genome sequence of *Aedes aegypti*, a major arbovirus vector Science 2007 316 1703 4 10.1126/science.1138878 17510324 PMC2868357

[B2] Terenius O Marinotti O Sieglaff D James AA Molecular genetic manipulation of vector mosquitoes Cell Host Microbes 2008 4 417 423 10.1016/j.chom.2008.09.002 PMC2656434 18996342

[B3] Dissanayake SN Marinotti O Ribeiro JMC James AA angaGEDUCI: Anopheles gambiae gene expression database with integrated comparative algorithms for identifying conserved DNA motifs in promoter sequences BMC Genomics 2006 7 116 10.1186/1471-2164-7-116 16707020 PMC1524951

[B4] Marinotti O Nguyen QK Calvo E James AA Ribeiro JMC Microarray analysis of genes showing variable expression following a bloodmeal in *Anopheles gambiae* Insect Mol Biol 2005 14 365 373 10.1111/j.1365-2583.2005.00567.x 16033430

[B5] Marinotti O Calvo E Nguyen QK Dissanayake S Ribeiro JMC James AA Genome-wide analysis of gene expression in adult *Anopheles gambiae* Insect Mol Biol 2006 15 1 12 10.1111/j.1365-2583.2006.00610.x 16469063

[B6] Ribeiro JM Arcà B Lombardo F Calvo E Phan VM Chandra PK Wikel SK An annotated catalogue of salivary gland transcripts in the adult female mosquito, *Aedes aegypti* BMC Genomics 2007 8 6 10.1186/1471-2164-8-6 17204158 PMC1790711

[B7] Brackney DE Isoe J WCB Zamora J Foy BD Miesfeld RL Olson KE Expression profiling and comparative analyses of seven midgut serine proteases from the yellow fever mosquito, *Aedes aegypti* J Insect Physiol 2010 56 736 744 10.1016/j.jinsphys.2010.01.003 20100490 PMC2878907

[B8] Kokoza VA Martin D Mienaltowski MJ Ahmed A Morton CM Raikhel AS Transcriptional regulation of the mosquito vitellogenin gene via a blood meal-triggered cascade Gene 2001 274 47 65 10.1016/S0378-1119(01)00602-3 11674997

[B9] Bailey TL Elkan C Fitting a mixture model by expectation maximization to discover motifs in biopolymers Proceedings of the Second International Conference on Intelligent Systems for Molecular Biology 1994 AAAI Press, Menlo Park, California 28 36 7584402

[B10] Hughes JD Estep PW Tavazoie S Church GM Computational identification of cis-regulatory elements associated with groups of functionally related genes in *Saccharomyces cerevisiae* Journal of Molecular Biology 2000 296 1205 1214 10.1006/jmbi.2000.3519 10698627

[B11] Roth FR Hughes JD Estep PE Church GM Finding DNA Regulatory Motifs within Unaligned Non-Coding Sequences Clustered by Whole-Genome mRNA Quantitation Nature Biotechnology 1998 16 939 945 10.1038/nbt1098-939 9788350

[B12] Cerenius L Söderhäll K The prophenoloxidase-activating system in invertebrates Immunol Rev 2004 198 116 126 10.1111/j.0105-2896.2004.00116.x 15199959

